# Global health funding and economic development

**DOI:** 10.1186/1744-8603-8-8

**Published:** 2012-04-10

**Authors:** Greg Martin, Alexandra Grant, Mark D'Agostino

**Affiliations:** 1 UNITAID, World Health Organization, 20 Avenue Appia, Geneva 27 1211, Switzerland; 2 Department of Clinical Pharmacology and Translational Medicine, Experimental Therapeutics Branch, Walter Reed Army Institute of Research, 503 Robert Grant Avenue, Silver Spring, MD 20910, USA

**Keywords:** Economic growth, GDP, Donor aid, Funding

## Abstract

The impact of increased national wealth, as measured by Gross Domestic Product (GDP), on public health is widely understood, however an equally important but less well-acclaimed relationship exists between improvements in health and the growth of an economy. Communicable diseases such as HIV, TB, Malaria and the Neglected Tropical Diseases (NTDs) are impacting many of the world's poorest and most vulnerable populations, and depressing economic development. Sickness and disease has decreased the size and capabilities of the workforce through impeding access to education and suppressing foreign direct investment (FDI). There is clear evidence that by investing in health improvements a significant increase in GDP per capita can be attained in four ways: Firstly, healthier populations are more economically productive; secondly, proactive healthcare leads to decrease in many of the additive healthcare costs associated with lack of care (treating opportunistic infections in the case of HIV for example); thirdly, improved health represents a real economic and developmental outcome in-and-of itself and finally, healthcare spending capitalises on the Keynesian 'economic multiplier' effect. Continued under-investment in health and health systems represent an important threat to our future global prosperity. This editorial calls for a recognition of health as a major engine of economic growth and for commensurate investment in public health, particularly in poor countries.

## Introduction

The argument for investing in public health initiatives and infrastructure in poor countries as presented in this editorial is twofold. Firstly, an increase in funding to address easily preventable deaths in poor countries would be consistent with our collective moral inclination. We seem however to be paralyzed by collective and institutional decision-making that fails to reflect the values we hold as individuals. And secondly, funds provided for public health spending in poor countries may translate into economic gains exceeding the initial investment.

## Group decision-making: Five million drowning

About ten million children die in low-income countries, defined as gross national income (GNI) less than USD $1,005 using the World Bank Atlas Method, every year. Millions of deaths are easily preventable with relatively inexpensive medicines and simple interventions like providing access to bed nets and clean drinking water. Furthermore, more than a thousand women die every day from preventable causes during pregnancy and childbirth--99% of these are in developing countries. The list of alarming statistics goes on, but most startling is the extent to which global death and disability from preventable causes can be efficiently and cost-effectively mitigated if we only had the political will to do so.

Well, what's stopping us? We propose that despite the recent economic crisis and the burgeoning era of austerity, the primary issue is not one of scarcity of resources, but rather a problem of collective decision-making.

As individuals, none of us would neglect to save a child drowning in a pool of water in front of us. In fact, when making the decision as an individual, each of us is likely to make considerable personal sacrifices in order to save the life of that child. Making that same decision as a group however--to decide as the collective two billion upper-middle and high income people living on the planet--that we will make the relatively small sacrifice needed to save the greater than five million children that die of preventable causes every year, is more challenging. There seem to be important differences in the way we make prioritize and decisions as individuals and the way we make decisions as a group.

Broadly speaking, humans subdivide into a myriad of groups: countries, gender groups, races, religious affiliations, cultural and socio-economic strata, and the like. At any one time, an individual is a member of multiple groups, each with an agenda and set of associated demands driven by incentives. Politicians, to survive the next election cycle, are incentivized to spend and allocate resources in such a way as to get re-elected by the collective group. Business executives are incentivized to maximize the utility of their company shareholders to maintain their employment and income. Academics are often incentivized to align their research focus with the priorities of the main funding organizations in their respective fields. And the majority, despite having genuinely altruistic impulses, engage in collective thought suppression, and the concern for children suffering in "remote places" is lost in the incessant pace and priorities of daily life.

And so it is that, despite being predominately good and empathetic individuals, we are unable to translate that individual impulse and unquestioning self-sacrifice to save a child dying in front of us into the scale of empathy and action that needs to be taken by us as a group. A lack of access to capital or funding is not the main driver towards this global issue, while many assume that is the root cause. The coordination of our collective decision making to ensure that it reflects the sum of our individual values is what is lacking. Collectively we do not prioritize health, even as our moral compass directs us towards investing in health and a positive correlation between healthcare expenditure and GDP has been demonstrated time and again.

## Healthcare funding and economic gains

In the words of Robert F. Kennedy, GNP "does not allow for the health of our children, the quality of their education, or the joy of their play. It does not include the beauty of our poetry or the strength of our marriages; the intelligence of our public debate or the integrity of our public officials.... It measures everything, in short, except that which makes life worthwhile." The pain associated with the loss of a loved one and the suffering associated with illness is difficult to quantify and so is mostly understated in the discussion surrounding the economic benefit of investing in health [1]. While the positive effect that improved health on the GDP of developing countries will be discussed in more detail below, we assert that combating disease should also be recognized as an economic end in-and-of itself [2].

In the context of the recent global financial crisis and subsequent austerity measures, investment in development by multilateral donors and donor countries needs to be prudent and focus on value for money. A tendency to cut back on spending on health in poor countries has translated into major budgetary cuts at the World Health Organization, the Global Fund, PEPFAR and other major donor agencies. The cancellation of The Global Fund to Fight Aids, Tuberculosis, and Malaria (GFATM) round 11 funding for example will have a clear and predictable impact on access to healthcare for millions of people in poor countries. A less obvious effect however will be the long-term impact on economic growth and poverty reduction in those countries. In this editorial we argue for an increased awareness of the positive effect of investing in health on the economic development.

The interconnectedness of the global economy extends far deeper than the superficial realm of trade and industry, and encompasses an interdependent ecology of our social, political, demographic and physical reality. Health and wellbeing forms an integral component of that ecology, and the promotion of health in developing countries represents more than just an opportunity to improve the physical lives of the poor, but indeed forms an essential rung without which our climb to global prosperity and security will be logistically impractical, economically inefficient and morally indefensible.

## Economic growth and health

Economic growth and the financial prosperity of a nation are proven to have a positive effect on population health. The causative paths that lead from increased wealth to improvements in health are well understood and broadly recognised. Populations with greater economic opportunities tend to have ready access to quality healthcare, less exposure to environmental hazards, better access to clean water, and improved opportunities to develop better preventative behaviour patterns [3]. This is perhaps most starkly illustrated by the changes seen in life expectancy following the industrial revolution, and the more recent improvements in child survival in West Africa.

While being richer does lead to health improvements, it is also true that there is a causative relationship in the other direction too. Health improvements lead to increased wealth and poverty reduction in four ways: Firstly, healthier populations are more economically productive; secondly, proactive healthcare leads to decrease in many of the additive healthcare costs associated with lack of care (treating opportunistic infections in the case of HIV for example); thirdly, improved health represents a real economic and developmental outcome in-and-of itself and finally, healthcare spending capitalises on the Keynesian 'economic multiplier' effect.

Employed healthcare workers spend their salaries on goods and services across multiple sectors of the economy, and public sector healthcare expenditures are often spent purchasing healthcare equipment and other goods and services from the private sector. The recipients of this spending, in turn, spend money across multiple sectors in the economy, and so on. Ultimately, this leads to an increase in the aggregate demand in the economy as a whole; this is particularly powerful if the original money comes from an outside source, like a multilateral donor. It is worth noting that this economic multiplier effect is lost or reduced where donor fund restrictions are in place, requiring that a significant portion of the donor funds be spent on goods and services provided by suppliers from the donor country. In the case of the Mozambique floods in 2000, medical volunteers had to buy Harley Davidson motorbikes, as these were the only US-made bikes that were available at short notice [4]. Donor fund restrictions might be significantly reduced as donor countries increasingly recognise the potential of both providing immediate aid and contributing to the increase in aggregate demand within the local economy within which they are working.

This bidirectional causative relationship between economic growth and health improvement presents an opportunity for a self-enforcing spiral that perpetuates itself either upwards or downwards. Missed opportunities to invest in cost-effective measures to combat disease at a population level should therefore be recognized as a major threat to health and development in general.

In the USA it has been estimated that the increase in life expectancy between 1970 and 2000 contributed an additional USD 3.2 trillion per year to the national economy (after accounting for increased healthcare costs during that period) [5]. In fact half of the overall economic growth in the USA during the last century can be attributed to improvements in health [6], as for every additional year of education attained through improved health status, a 15% higher starting wage and a doubling of the rate of subsequent salary increase was attained [3]. In poor countries, a 40% increase in life expectancy is associated with a 1.4% increase in GDP per capita, and malnutrition world-wide impacts global GDP negatively by up to 4.7% [7]. Half the difference between the rate of economic growth of the least developed nations in Africa and that of the high-growth countries of East Asia can be ascribed to a combination of disease, demography and geography [3].

## Child mortality and human capital

In poor countries, improvements in child mortality rates ultimately result in a reduced birth rate, as families no longer perceive the need to compensate for high infant mortality by having more children. This translates into a higher parental investment per child in health and education [7]. Importantly, reduced child mortality also circumvents the economic losses associated with having no return on the investment in a child's education in the form of economically productive activity when the child becomes an adult. This was recognized as far back as 1842 when Sir Edwin Chadwick argued in his report on *The Sanitary Condition of the Labouring Population *that investment in sanitation would reduce economic losses created by the early death of poor children [3].

The output of human capital can be thought of as a function of productive time; it has been shown that the economic gains from improved health equal or exceed those due to an increase in education and on-the-job training [3]. The Nobel Laureate Theodore Schultz states that human capital is "vastly larger than all other forms of wealth taken together" [8]. Importantly, for many poor people, their only asset (for economic productivity) is their body and the economic shock associated with illness can drive them into abject poverty [7].

## Neglected tropical diseases

One of the most glaring opportunities for exploiting the relationship between investing in health and economic development is that of investing in neglected tropical diseases (NTDs), which include 7 helminth infections (hookworm, trichuriasis, ascariasis, schistosomiasis and dracunculiasis, onchocerciasis and lymphatic filariasis), 3 protozoan infections: (leishmaniasis, trypanosomiasis and Chagas' disease) and 3 bacterial infections (leprosy, trachoma and Buruli ulcer). Many of the names of these pathogens may be unfamiliar to the reader, but collectively these diseases contribute toward a large proportion of the burden of disease in poor countries, and significantly impact economic growth and development in countries where they are endemic.

More than a million children in Africa are "polyparasitized", infected with both malaria and a combination of these NTDs [9], leading to lower school attendance, lethargy and impaired attention as well as having indirect (though malabsorption of nutrients and iron-deficiency anaemia) and direct effects on the brain [10]. Schistosomiasis is associated with poor short-term memory and slower reaction times in children [11]. The eroded economic opportunities for these children manifests in an estimated drop in later income by as much as 17% [12]. For the equivalent of just two days of Pentagon spending (about USD 3 billion at the time of writing) a year, comprehensive Africa-wide control of both malaria and the neglected tropical diseases (NCDs) could be achieved [9].

## HIV, TB and malaria

The Global Fund, together with PEPFAR, UNITAID, CHAI, GAVI, the World Bank, the World Health Organization (WHO) and others have made significant inroads toward making treatment for these diseases both available and affordable. The goal of the WHO, UNIAID and UNICEF to virtually eliminate Mother to Child Transmission (MTCT) of HIV by 2015 illustrates the level of global commitment to improve the health of the poor. However, the combined research into new treatments for these diseases that represent over 90% of the global burden of disease still amounts to less than 10% of the global investment in health research.

A 10% reduction of malaria in endemic areas has been shown to be associated with a 0.3% increase in economic growth [13] while the direct and indirect costs of malaria in sub-Saharan Africa consume about 0.6% of the GDP in the region [14]. Treatment costs for an existing cohort of 3.5 million HIV positive patients on ART (based on 2009 prices) for the next 10 years would cost about $14.2 billion but is expected to have a net economic benefit of between $12 billion and $34 billion through increased productivity, averted orphan care, deferred medical treatment for opportunistic infections and end of life care [15].

## Conclusion

To divert public spending in poor countries from health with the belief that if these nation becomes prosperous, their health problems will take care of themselves, is a mistake. Under investment in health and health systems represent an important threat to our future global prosperity. This paper calls for a recognition of health as a major engine of economic growth and for commensurate investment in public health, particularly in poor countries.

## Competing interests

The authors declare that they have no competing interests.
